# Supplementary material for: CELLECT: contrastive embedding learning for large-scale efficient cell tracking
Source: Nat Methods. 2025 Oct 20;22(11):2411–22. doi: 10.1038/s41592-025-02886-x (PMC12615263; doi:10.1038/s41592-025-02886-x)
Supplement: Supplementary file 1 — Supplementary Figs. 1–20 and Tables 1–4 and Captions for Supplementary Videos 1–4. [file 41592_2025_2886_MOESM1_ESM.pdf]

nature methods

Supplementary information https://doi.org/10.1038/s41592-025-02886-x

CELLECT: contrastive embedding learning for large-scale efficient cell tracking

In the format provided by the authors and unedited

Supplementary Information

Contrastive Embedding Learning for Large-scale Efficient Cell Tracking

Hongyu Zhou, Seonghoon Kim, Zhifeng Zhao, Jiaqi Fan, Wen Huang, Xinghua Sui, Lizhi Shao, Haoran An, Jing-Ren Zhang, Jiamin Wu, Qionghai Dai

I. Supplementary Figures

**Supplementary Figure 1**	Detailed network framework of CELLECT
**Supplementary Figure 2**	Examples of the output confidence map of cell centers obtained by CELLECT
**Supplementary Figure 3**	Clustering of feature vectors obtained by CELLECT via PCA across the adjacent frames
**Supplementary Figure 4**	Visualization of the embedding map obtained by CELLECT
**Supplementary Figure 5**	Illustrations of the intra-frame MLP and inter-frame MLP
**Supplementary Figure 6**	Illustration of different types of errors during cell tracking used for evaluation
**Supplementary Figure 7**	Ablation study of CEN module on tracking performance
**Supplementary Figure 8**	Influence of level configuration during training on tracking performance
**Supplementary Figure 9**	Influence of patch size on tracking performance
**Supplementary Figure 10**	Influence of the search-mark structure on tracking performance
**Supplementary Figure 11**	Influence of imaging frame rate on tracking performance under different candidate selection strategies
**Supplementary Figure 12**	Influence of imaging frame rate on tracking performance under different training dataset and candidate numbers used
**Supplementary Figure 13**	Evaluation of the reproducibility of the model
**Supplementary Figure 14**	Evaluation of the reproducibility of cell linking during different imaging frame rate
**Supplementary Figure 15**	Cell tracking for dual-color labeled T cells

**Supplementary Figure 16**	Comparisons of the tracking results of B-cell dynamics obtained by CELLECT and Imaris
**Supplementary Figure 17**	Extracted traces of highly migratory B cells
**Supplementary Figure 18**	Tracking performance of CELLECT on terabyte-level imaging datasets of B-cell dynamics
**Supplementary Figure 19**	Influence of the intensity threshold on the segmentation results of Imaris with comparisons to CELLECT
**Supplementary Figure 20**	Continuous tracking and segmentation of a single neutrophil chasing and engulfing bacteria

II. Supplementary Tables

**Supplementary Table 1**	Quantitative results on mskcc-confocal and nih-ls data
**Supplementary Table 2**	Quantitative results for each fold in the cross-validation experiments on mskcc-confocal and nih-ls data
**Supplementary Table 3**	Input and output tensor dimensions for each module
**Supplementary Table 4**	Quantitative ablation study of individual modules in the CELLECT framework

III. Supplementary Videos

**Supplementary Video 1**	Confidence map generated by CELLECT on a dataset of membrane-labeled cells
**Supplementary Video 2**	Efficient cell tracking of CELLECT on *C. elegans* dataset compared with Linajea
**Supplementary Video 3**	3D cell tracking of large-scale B cells during the formation of germinal center
**Supplementary Video 4**	Continuous tracking of neural activities at single-cell resolution in *Drosophila* brain during tissue deformation

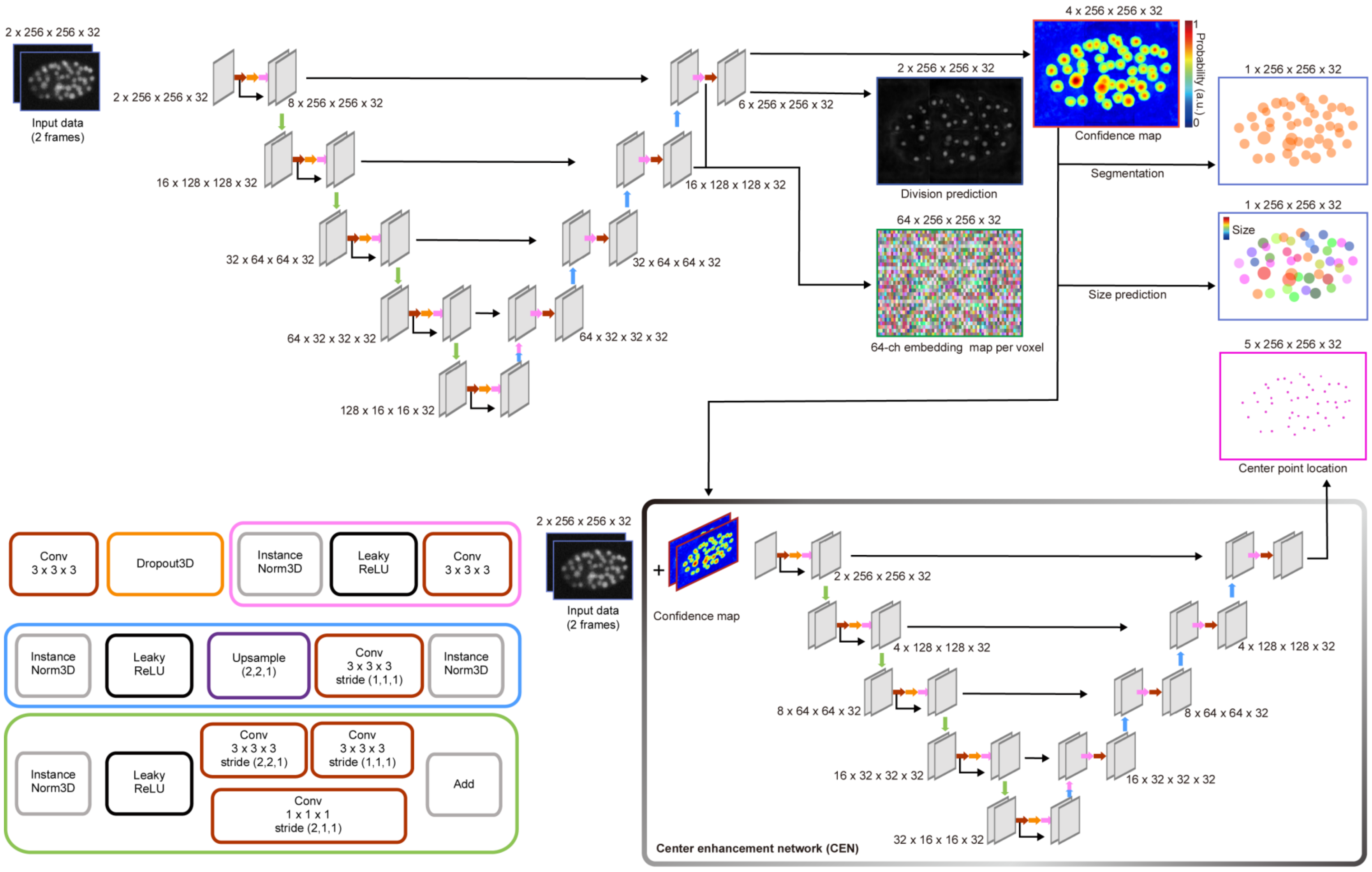
2 x 256 x 256 x 32
Input data
(2 frames)
2 x 256 x 256 x 32
8 x 256 x 256 x 32
16 x 128 x 128 x 32
32 x 64 x 64 x 32
64 x 32 x 32 x 32
128 x 16 x 16 x 32
64 x 32 x 32 x 32
32 x 64 x 64 x 32
16 x 128 x 128 x 32
6 x 256 x 256 x 32
4 x 256 x 256 x 32
Confidence map
1
Probability (a.u.)
0
2 x 256 x 256 x 32
Division prediction
64 x 256 x 256 x 32
64-ch embedding map per voxel
1 x 256 x 256 x 32
Segmentation
1 x 256 x 256 x 32
Size
Size prediction
5 x 256 x 256 x 32
Center point location
Conv
3 x 3 x 3
Dropout3D
Instance
Norm3D
Leaky
ReLU
Conv
3 x 3 x 3
Instance
Norm3D
Leaky
ReLU
Upsample
(2,2,1)
Conv
3 x 3 x 3
stride (1,1,1)
Instance
Norm3D
Instance
Norm3D
Leaky
ReLU
Conv
3 x 3 x 3
stride (2,2,1)
Conv
3 x 3 x 3
stride (1,1,1)
Conv
1 x 1 x 1
stride (2,1,1)
Add
2 x 256 x 256 x 32
Input data
(2 frames)
+
Confidence map
2 x 256 x 256 x 32
4 x 128 x 128 x 32
8 x 64 x 64 x 32
16 x 32 x 32 x 32
32 x 16 x 16 x 32
16 x 32 x 32 x 32
8 x 64 x 64 x 32
4 x 128 x 128 x 32
Center enhancement network (CEN)

Supplementary Figure 1 | Detailed network framework of CELLECT. The overall framework of the CELLECT model is illustrated; The upper-left section depicts the main network structure, a 3D U-Net, with data dimensions and arrows representing the data flow and connection between modules. The color of the arrows corresponds to the network modules shown in the at the left bottom panel. The main model generates 3 outputs: cell division predictions, a confidence map, and a 64-dimensional embedding map, all maintaining same spatial resolution as the input, derived from 2 frames of input images. The confidence map serves as the basis for cell segmentation and size estimation. The lower-right section introduces the Center Enhancement Network (CEN), a light-weight 3D U-Net model. This network takes the confidence map and the 2 original image frames as inputs, refining the probability distribution by focusing on high-probability regions around cell center points while suppressing probabilities in other areas. The peaks in the confidence map generated by the CEN correspond directly to precise center point locations. This framework enables high-accuracy cell division prediction, segmentation, and size estimation while significantly improving the precision of center point localization. The network outputs shown here were generated from the Cell Tracking Challenge (CTC) Fluo-N3DH-CE dataset. The model used was trained on sequence #3 of the mskcc-confocal dataset.

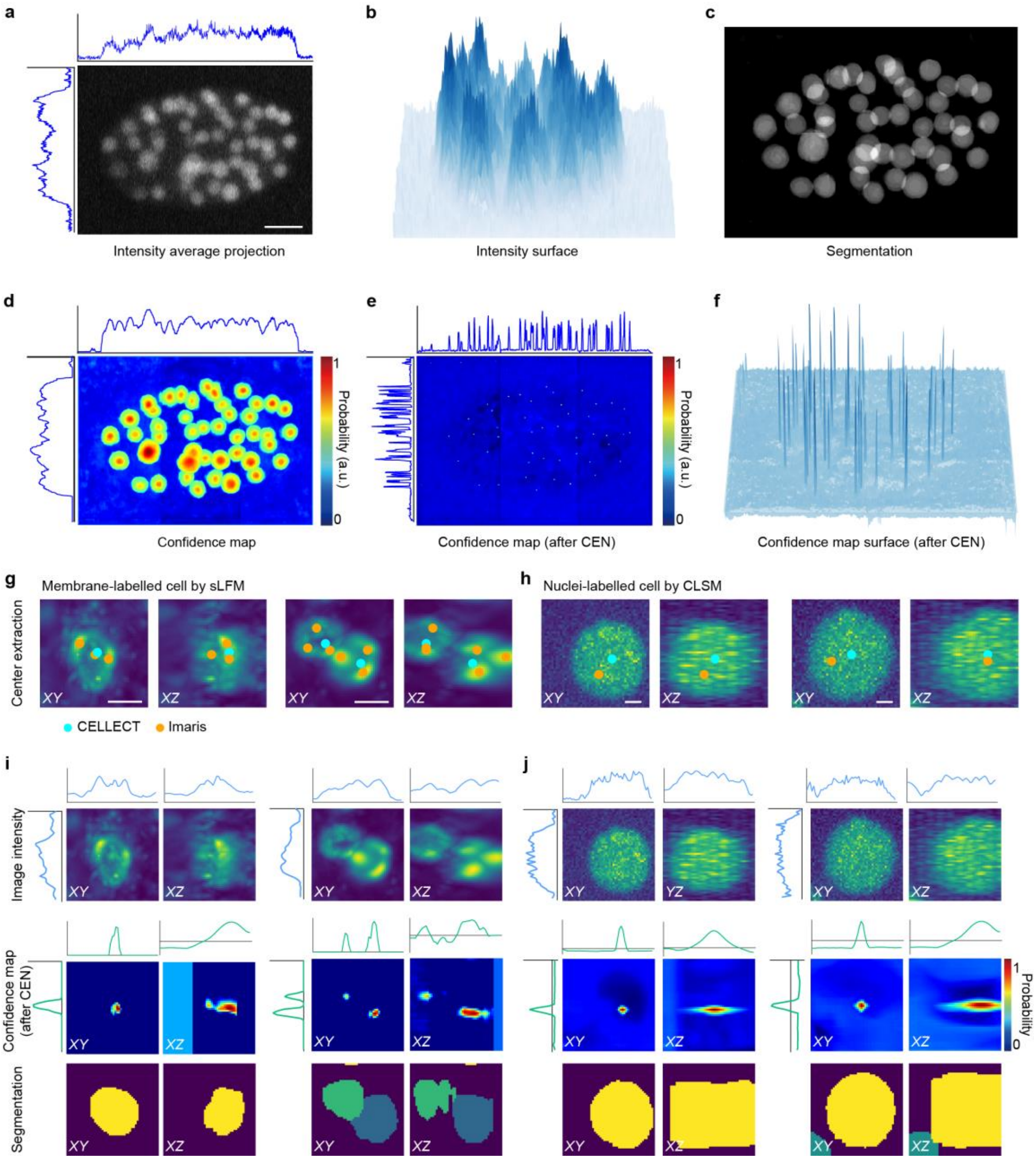

Supplementary Figure 2 | Examples of the output confidence map of cell centers obtained by CELLECT. a-b, Visualization of the intensity distribution of the example data. (**a**) 2D average projected image of the intensity distribution of input image. (**b**) 3D visualization of the intensity surface. **c**, Cell segmentation regions generated by the CELLECT model. **d-e**, 2D maximum projected image of the confidence map (**d**) before and (**e**) after CEN. **f**, 3D visualization of the confidence map surface after CEN. **g-h**, Comparison of center extraction performance between CELLECT and Imaris for (**g**) membrane-labeled cells image acquired by using Scanning Light-Field Microscopy (sLFM) and (**h**) nuclei-labeled cells imaged using Confocal Laser Scanning Microscopy (CLSM). **i-j**, Input image intensity, confidence maps (after CEN), and segmentation results produced by the CELLECT model. Panels (**a–f**) use data from the Cell Tracking Challenge (CTC) Fluo-N3DH-CE dataset. Panels (**g–j**) use nuclei-labeled data from the mskcc-confocal dataset and membrane-labeled neutrophil data from mouse imaging, as also shown in **Fig. 4**. All

results were generated using a CELLECT model trained on sequence #3 of the mskcc-confocal dataset. In panel (**g**) and (**i**), the membrane-labeled structure in the right-hand cell is confirmed to correspond to a single cell. This is supported by a continuous, closed membrane signal without interruption, indicating that it is not the result of overlapping or adjacent cells. Scale bars: 10 µm (**a**), 5 µm (**g**), 1 µm (**h**).

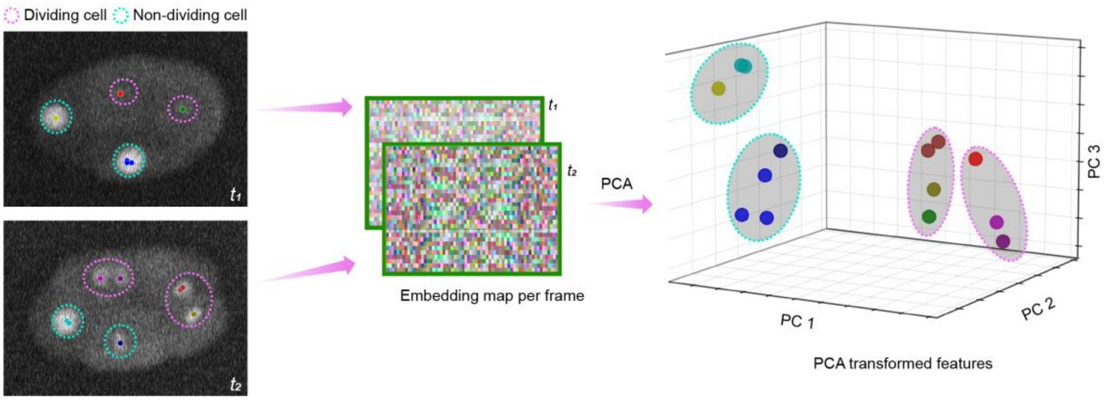

Supplementary Figure 3 | Clustering of feature vectors obtained by CELLECT via PCA across the adjacent frames. From 2 adjacent frames of input images, we extracted an embedding map for each frame and applied Principal Component Analysis (PCA) to reduce the dimensionality of feature vectors. In the resulting 3D PCA space, feature vectors corresponding to the same cell identity across the adjacent frames formed distinct clusters. Even dynamical changes during the division process, the feature distances of the daughter cells remained relatively close to that of the parent cell. By combining with Euclidean distances between cell centers, we inferred the potential division relationships between cells. The example shown here uses data from the Cell Tracking Challenge (CTC) Fluo-N3DH-CE dataset. The model used for feature extraction was trained on sequence #3 of the mskcc-confocal dataset.

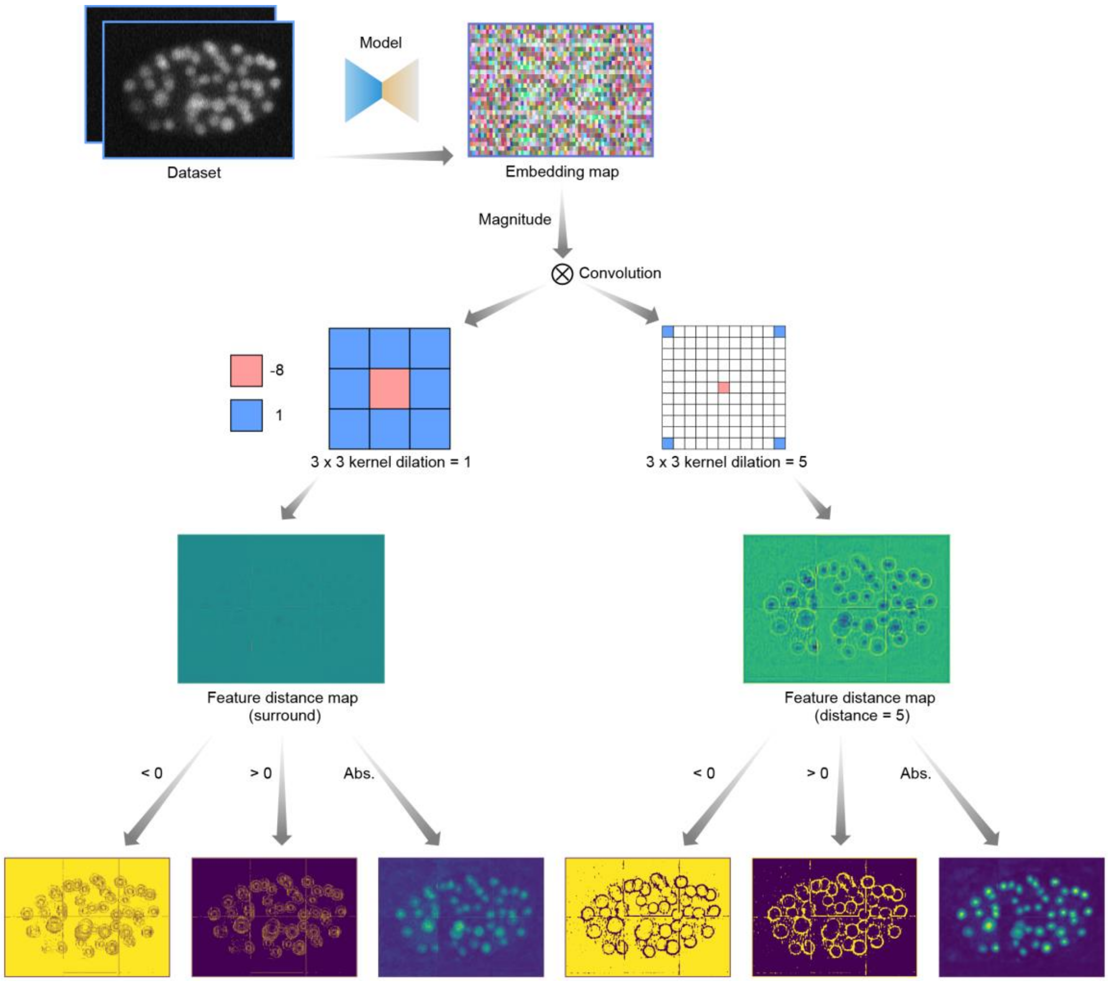

Supplementary Figure 4 | Visualization of the embedding map obtained by CELLECT. To analyze the spatial patterns captured by the model, we extracted embedding map corresponding to each voxel and computed their differences with surrounding voxels. This was achieved by convolving the squared values of the embedding map with a dilated convolution kernel, simulating neighborhood interaction. A dilation rate of 1 represents the average difference with adjacent voxels, while a dilation rate of 5 represents the difference with voxels at a distance of 5 voxels in all directions. The resulting feature distance map highlights the magnitude and positive or negative of feature differences between distinct cell regions in the image. At the boundaries, the opposing patterns between the central and surrounding areas particularly emphasize the differences in feature values between cellular and non-cellular regions. These differences can also assist in optimizing cell segmentation during the inference phase, especially under sparse annotation conditions. The artifacts observed at patch boundaries arise from the independent inference of each patch, which do not affect the accuracy of segmentation or tracking, as only the embedding vectors at predicted cell centers are used for downstream analysis, and the boundary variations are negligible. The visualization shown here is based on the Cell Tracking Challenge (CTC) Fluo-N3DH-CE dataset. The model used was trained on sequence #3 of the mskcc-confocal dataset.

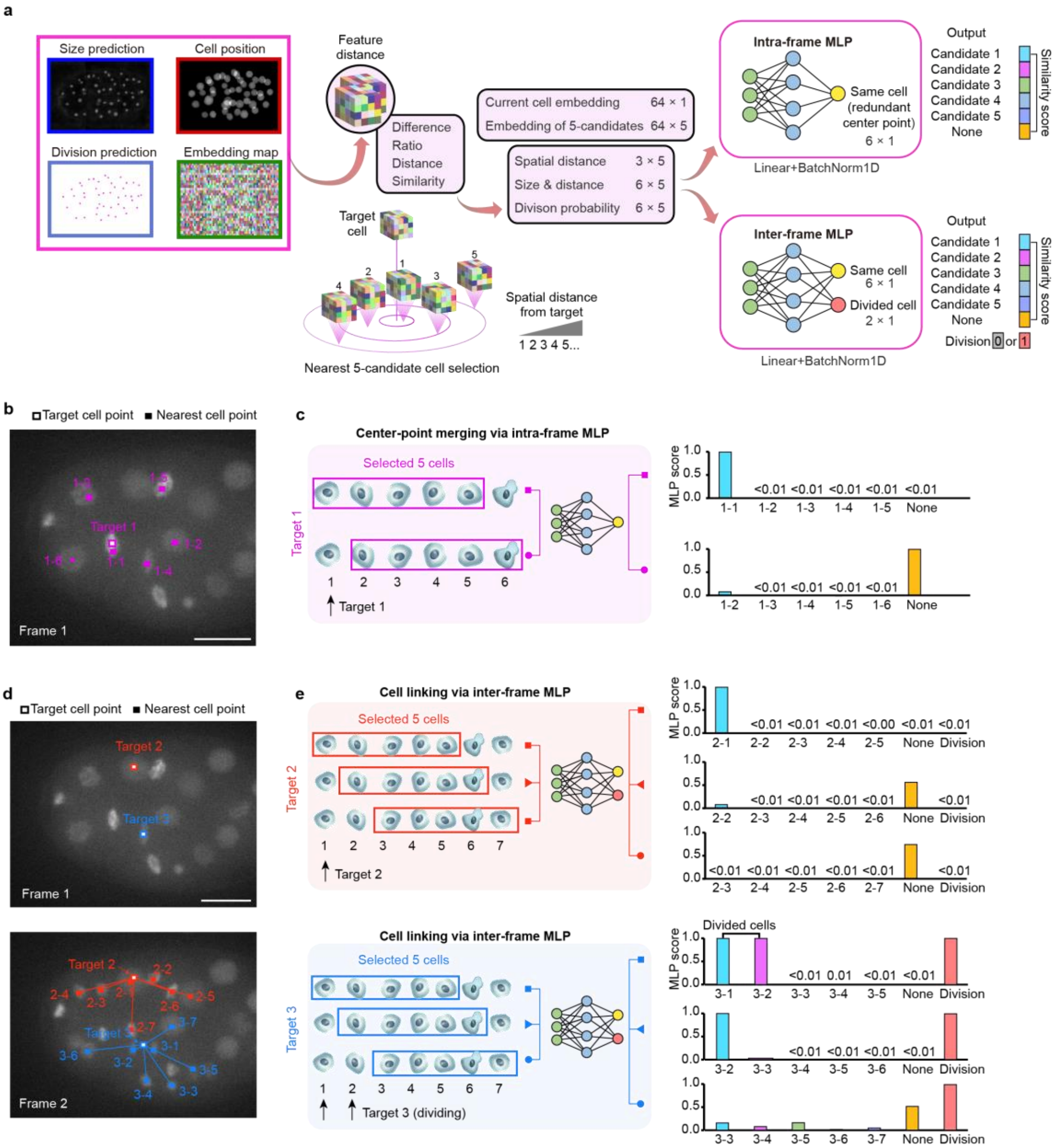

Supplementary Figure 5 | Illustrations of the intra-frame MLP and inter-frame MLP. a, Illustration of the workflow of 2 MLPs. The 5 nearest candidate cells to the target cell are selected based on the spatial distances between cell centers. For intra-frame MLP, we selected the 5 candidate cells within the same time frame. For inter-frame MLP, we selected the 5 candidate cells in the next frame closest to the target cell in the current frame. For each candidate, four feature distances relative to the target cell are computed: (1) size and division probability difference, (2) size and division probability ratio, (3) Euclidean distance, and (4) feature similarity. These metrics serve as inputs to the MLPs. The intra-frame MLP identifies redundant cell center point detections of the same cell, while the inter-frame MLP determines whether the target cell matches a candidate in the next frame or undergoes division. Each MLP outputs 6 similarity scores including 5 candidates and one 'None' class indicating that the target cell is not matched with any of the 5 candidates. The inter-frame MLP outputs 6 similarity scores and also provides a division prediction. **b**, Representative example of intra-frame MLP. For a given target cell (target 1), its six nearest cell center points (labeled 1-1 to 1-6) are identified within the same frame. We note that point 1-1 is a redundant detection of the same cell as the target. **c**, Intra-frame MLP outputs for different combinations of 5

out of the 6 candidates. Including the redundant detection (1-1) results in high confidence scores for that match, whereas its exclusion leads to the 'None' class having the highest score. **d**, Inter-frame MLP example for two target cells: target 2 (not dividing) and target 3 (undergoing division). Each is associated with 7 nearest candidates in the next frame. **e**, Inter-frame MLP outputs using three different candidate selection sets (e.g., 1-5, 2-6, and 3-7) for target cells 2 and 3. Inclusion of the correct corresponding cell (e.g., 2-1 for target 2) or daughter cells (3-1 and 3-2 for target 3) in the candidate set results in high similarity scores from the MLP. Exclusion of true matches causes the 'None' score to dominate. The division prediction is mainly driven by the input division probability of the target cell, and is only marginally influenced by candidate selection. The visualization in (**a**) is based on the Cell Tracking Challenge (CTC) Fluo-N3DH-CE dataset, while the examples in (**b–e**) are from sequence #2 of the mskcc-confocal dataset. All results were generated using a model trained on sequence #3 of the mskcc-confocal dataset.

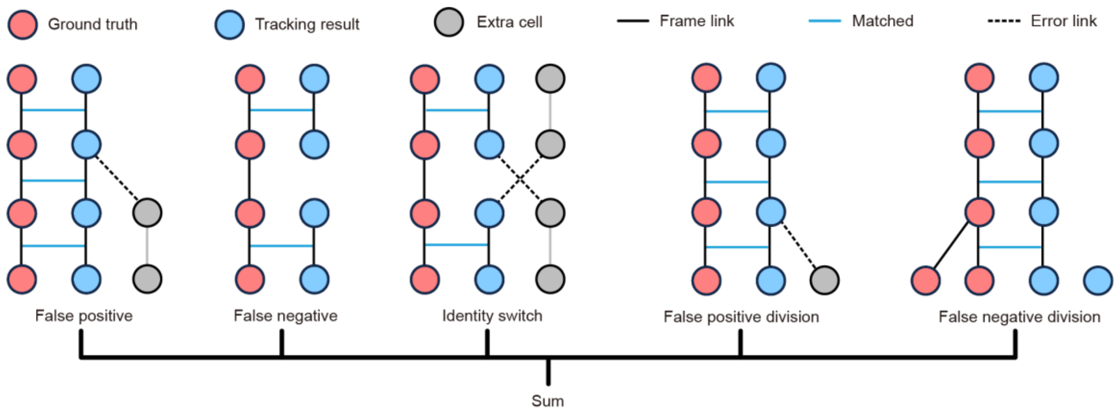

Supplementary Figure 6 | Illustration of different types of errors during cell tracking used for evaluation. False positive (FP), false negative (FN), identity switch (IS), false positive division (FP-D) and false negative division (FN-D). Red and blue circles indicate ground truth tracks and reconstructed tracks, respectively. Blue lines represent edges correctly matched between the ground truth and reconstructed track, while dashed lines highlight redundant error tracks.

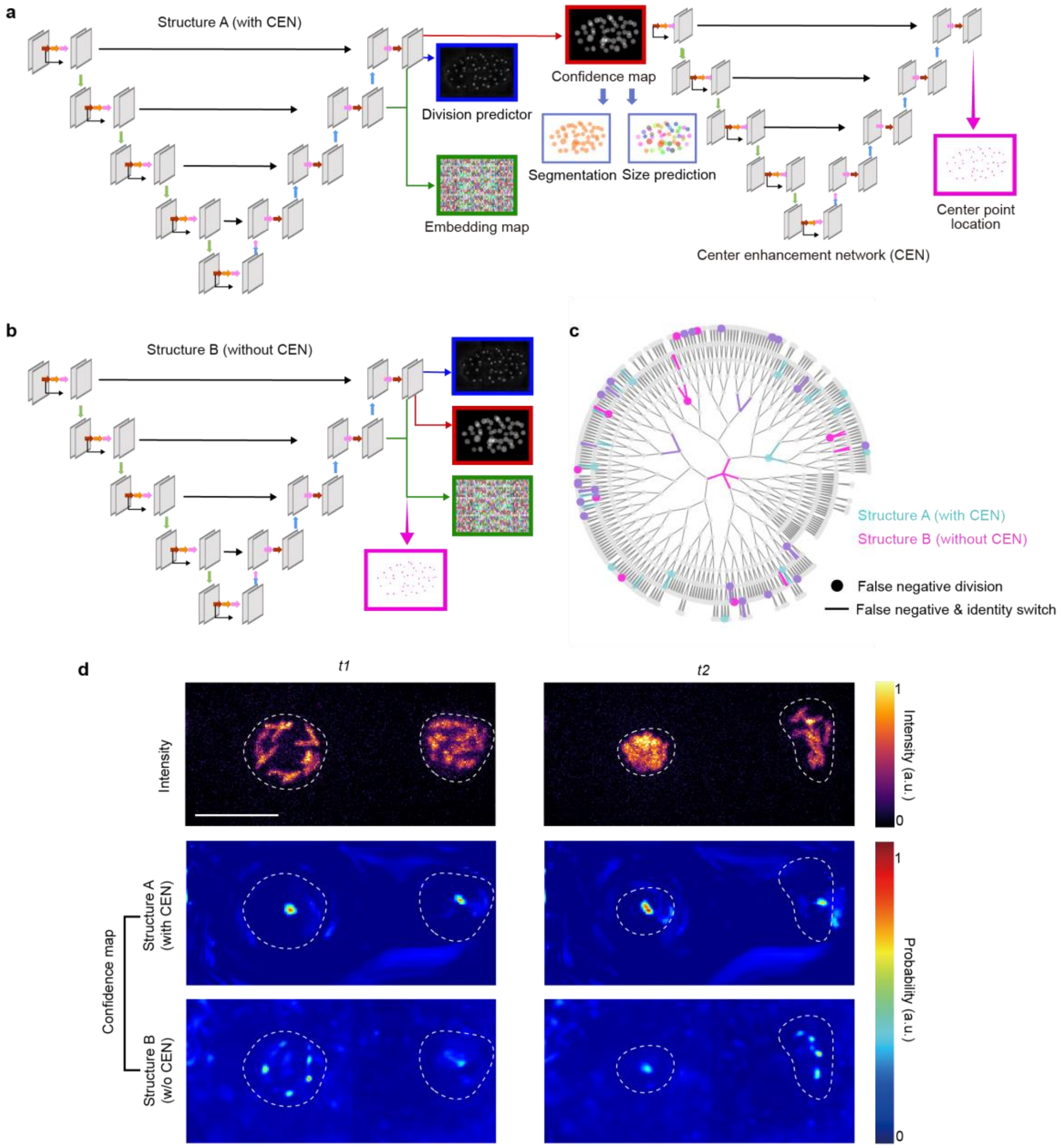

Supplementary Figure 7 | Ablation study of CEN module on tracking performance. a, The CELLECT model structure used in this study. **b**, A modified version of the CELLECT model without the CEN subnetwork. While the CEN structure was removed, its training methodology and output format were retained to mitigate performance drop. Without these modifications, simply removing the CEN would result in outcomes similar to the 2-level configuration output scenario, leading to significantly worse performance and making it difficult to compare model structures. **c**, We validated the performance differences of 2 methods on the mskcc-confocal dataset[1,2] #2. As shown, the model without the CEN module produces more errors, with incorrect links appearing even in the early stages. **d**, A comparison of the confidence maps of the 2 models in the early stages. We found that the CEN module shows more accurate performance of center extraction especially for larger cells, thus more efficiently reduces incorrect cell linking. All evaluation data in this figure were drawn from mskcc-confocal dataset #2. All models were trained using mskcc-confocal dataset #3. Scale bars: 10 μm.

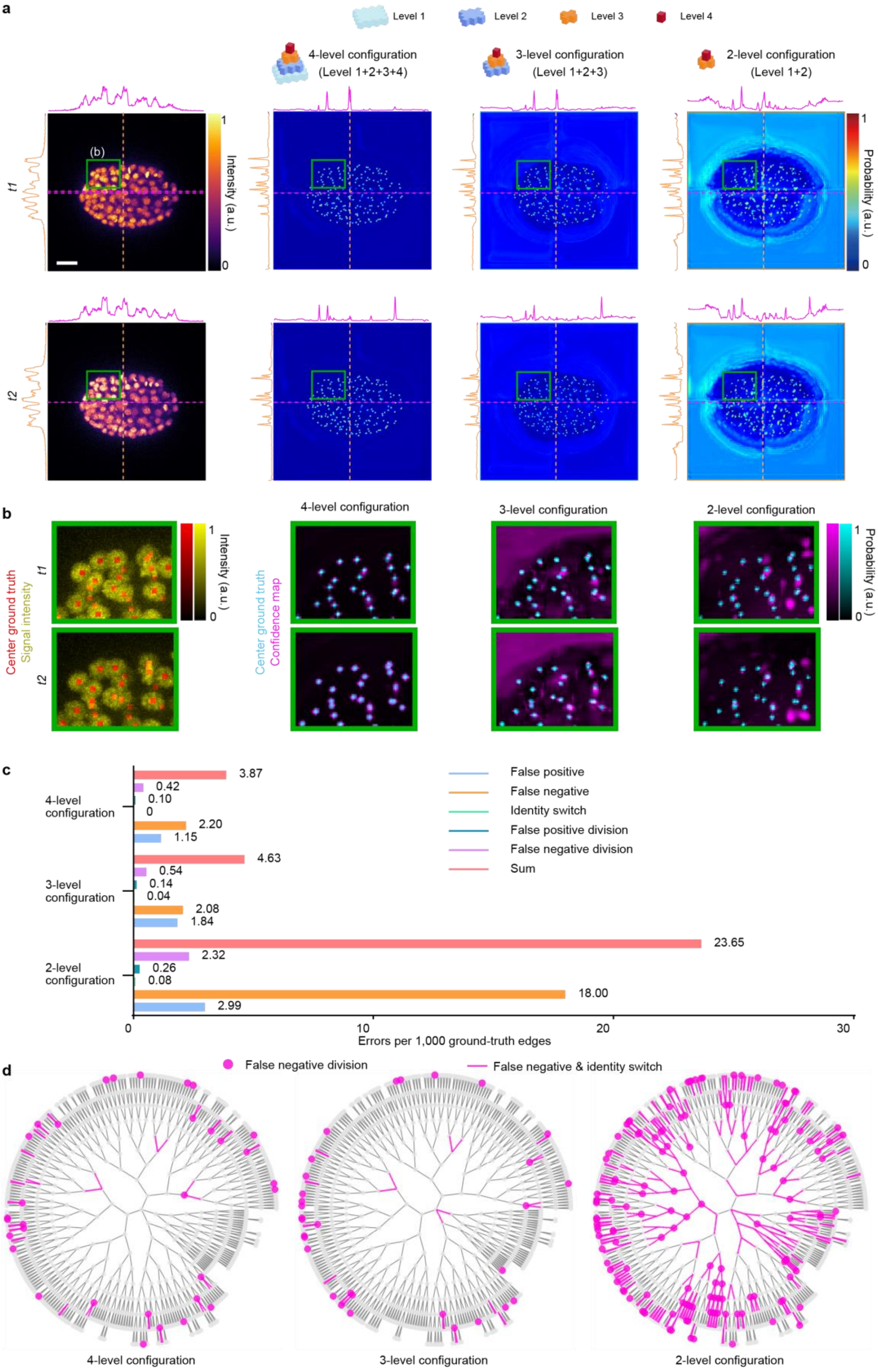

Supplementary Figure 8 | Influence of level configuration during training on tracking performance. a, 2 frames of signal intensity maps and confidence maps obtained with 3-different

level configurations via CELLECT. Horizontal and vertical line profiles are depicted in purple and orange, respectively. **b**, Extended view of ground truth-applied maps. From the ground truth of the center location of each cell, we generated a 3-voxel radius binary mask for the 2 frames and multiplied it with intensity maps and confidence maps, respectively. The location of the extended view is indicated with a green box in each original image in (**a**). Overlapped areas with ground truth are shown in red and cyan for intensity and confidence maps, respectively. **c-d**, The impact of level configuration on the model's performance was evaluated, demonstrating that the 4-level configuration used in this study achieved the best performance. This configuration enhances center point distinction through multi-level refinement. Error connections are highlighted in pink, while correct connections are depicted in grey. All evaluation data in this figure were drawn from mskcc-confocal dataset #2. All models were trained using mskcc-confocal dataset #3. Scale bars: 10 μm.

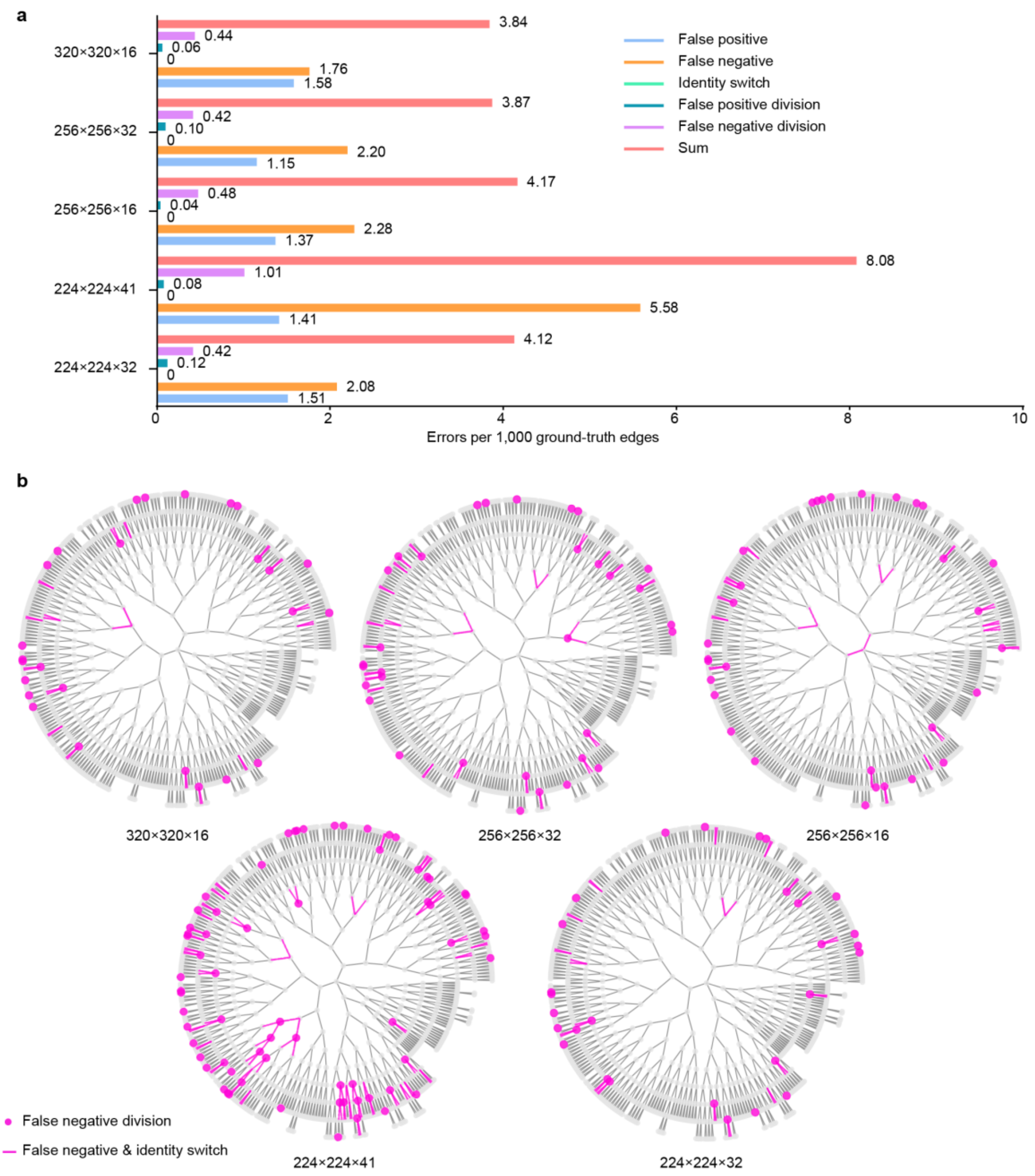

Supplementary Figure 9 | Influence of patch size on tracking performance. The impact of training size on the model's performance was evaluated, revealing that performance is primarily influenced by the training size and the axial resolution of the image. In general, larger training sizes lead to improved performance. However, for the 224×224×41 configuration (where the dataset contains 41 axial slices), the patch spans the entire z-axis, resulting in reduced variability in axial sampling during training and thus lower tracking accuracy. Error connections are highlighted in pink, while correct connections are depicted in grey. This data was retrained following the experiments presented in **Fig. 2**. All evaluation data in this figure were drawn from mskcc-confocal dataset #2. All models were trained using mskcc-confocal dataset #3.

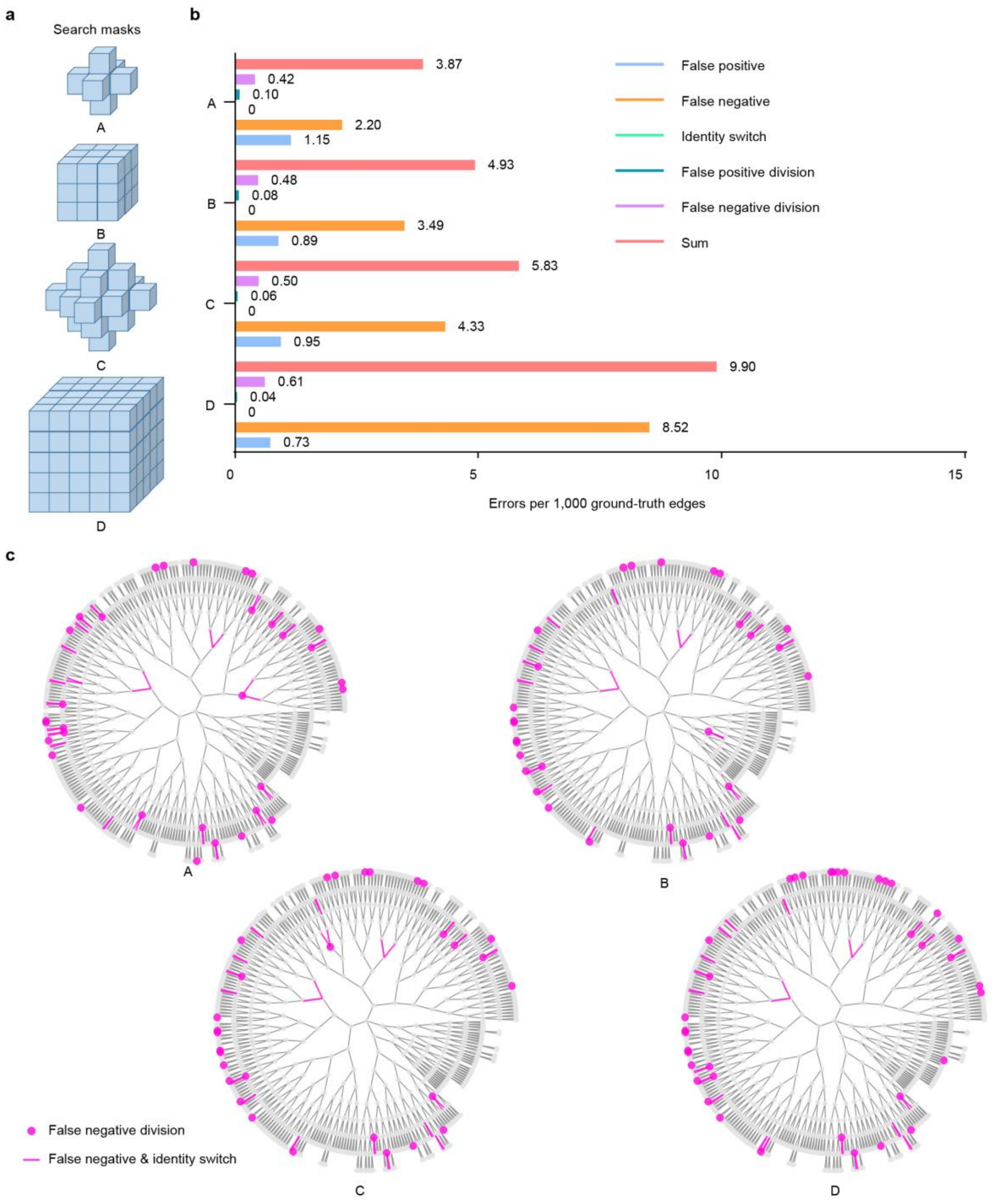

Supplementary Figure 10 | Influence of the search-mark structure on tracking performance. The impact of search mask structure on the model's performance was evaluated, revealing that performance is predominantly influenced by cell density and axial resolution. Among the 4 different search mask structures assessed, ensuring the absence of multiple center points within a mask's range was a key consideration. However, with very low axial resolution, larger search masks occasionally failed to detect certain center points. To address this, we adopted the most streamlined structure A. Error connections are highlighted in pink, while correct connections are depicted in grey. All evaluation data in this figure were drawn from mskcc-confocal dataset #2. All models were trained using mskcc-confocal dataset #3.

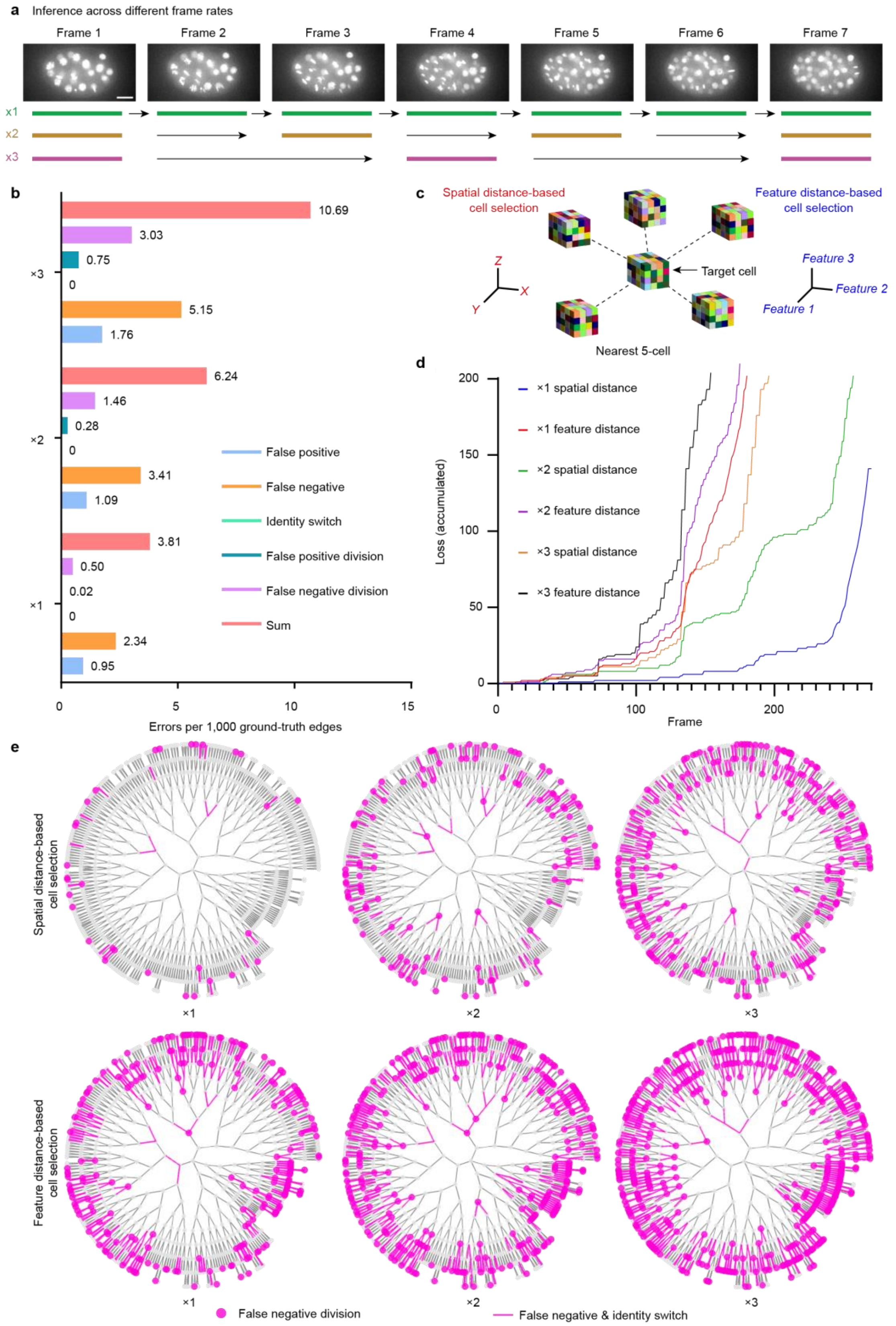

Supplementary Figure 11 | Influence of imaging frame rate on tracking performance under different candidate selection strategies. a, Illustration of 3 different levels of temporal down-sampling were used for evaluation: ×1 (75-s interval), ×2 (150-s interval), and ×3 (225-s interval) down-sampling. **b**, Quantitative tracking performance of CELLECT under 3 conditions of temporal

down-sampling. **c**, Schematic illustration of 2 cell selection strategies: left, spatial-distance-based selection of the 5 nearest neighbors (used in CELLECT); right, feature-distance-based selection of the 5 most similar feature vectors. **d**, Error curves (FN + FND + IS) over time for both strategies under different temporal down-sampling. The feature distance-based strategy performs comparably at early frames when cell density is low, even under reduced temporal resolution, but its performance degrades rapidly as cell density increases over time. **e**, Circular tree diagrams displaying tracking errors across all 6 settings. All evaluation data were drawn from mskcc-confocal dataset #2; the model was trained on mskcc-confocal dataset #3 without temporal down-sampling. Scale bar: 10 μm.

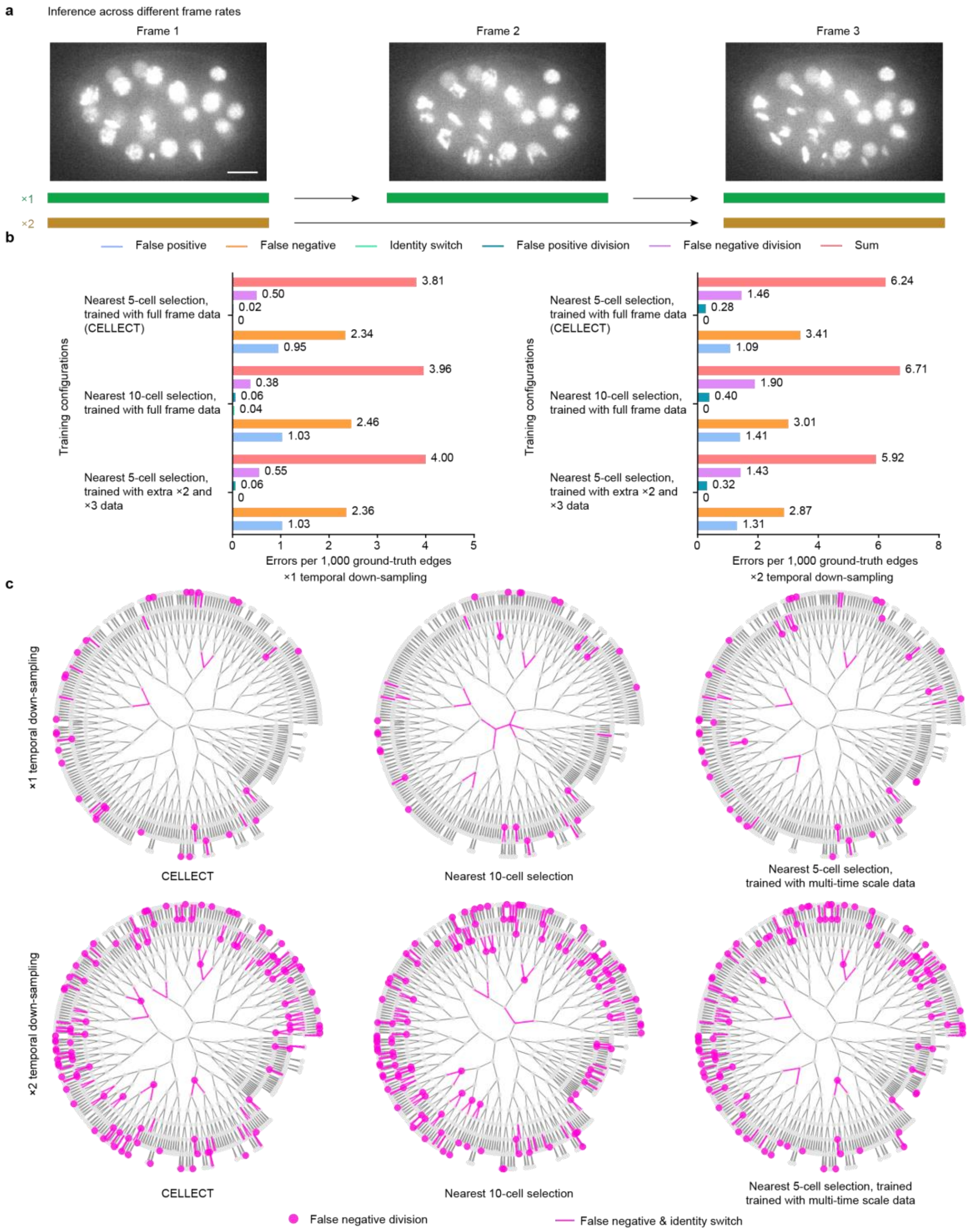

Supplementary Figure 12 | Influence of imaging frame rate on tracking performance under different training dataset and candidate numbers used. **a**, Illustration of the 2 temporal resolutions used in the inference process: ×1 (75 s) and ×2 (150 s) down-sampling. **b**, Tracking performance of 3 different configurations for different temporal down-sampling in inference: the default CELLECT configuration, model using 10 candidate cells, and model using 5 candidate cells but trained with different temporal resolutions (randomly incorporating dataset with ×1, ×2 and ×3 temporal down-sampling). All configurations show comparable performance under ×1 temporal down-sampling. However, under ×2 temporal down-sampling, models trained with an expanded candidate pool or mixed-temporal-resolution data exhibit reduced false negative (FN) rates, indicating improved robustness to lower temporal resolution. **c**, Circular lineage diagrams

corresponding to the 6 experimental settings. All results were drawn from mskcc-confocal dataset #2, with models trained on dataset #3 with and without temporal down-sampling. Scale bar: 10 μm.

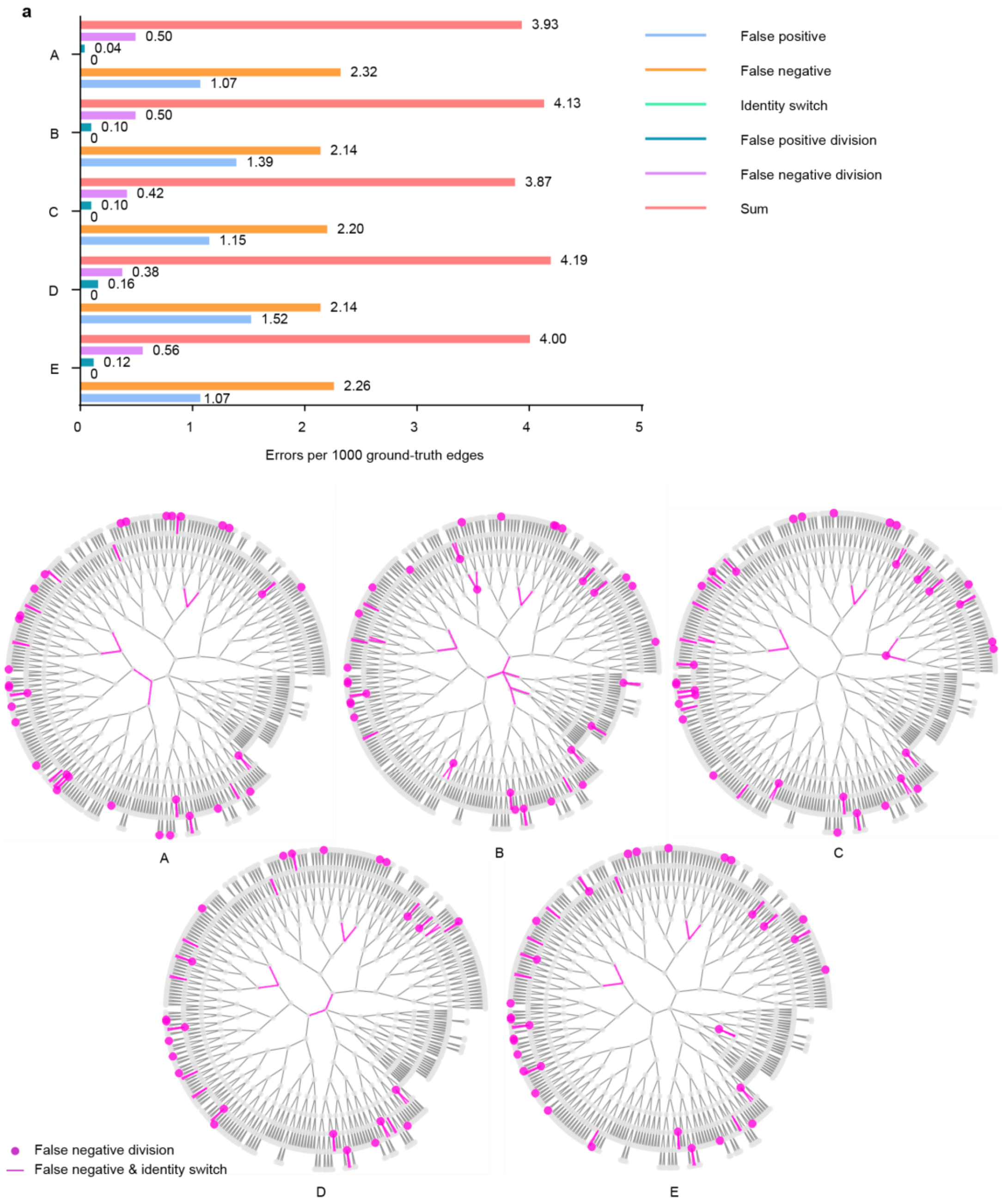

Supplementary Figure 13 | Evaluation of the reproducibility of the model. To assess the reproducibility of the model performance, we trained the model 5 times using identical parameters but with non-deterministic random sampling during training. The results revealed that, despite minor fluctuations, the model's overall performance remains highly consistent and reproducible. Error connections are highlighted in pink, while correct connections are depicted in grey. All evaluation data in this figure were drawn from mskcc-confocal dataset #2. All models were trained using mskcc-confocal dataset #3.

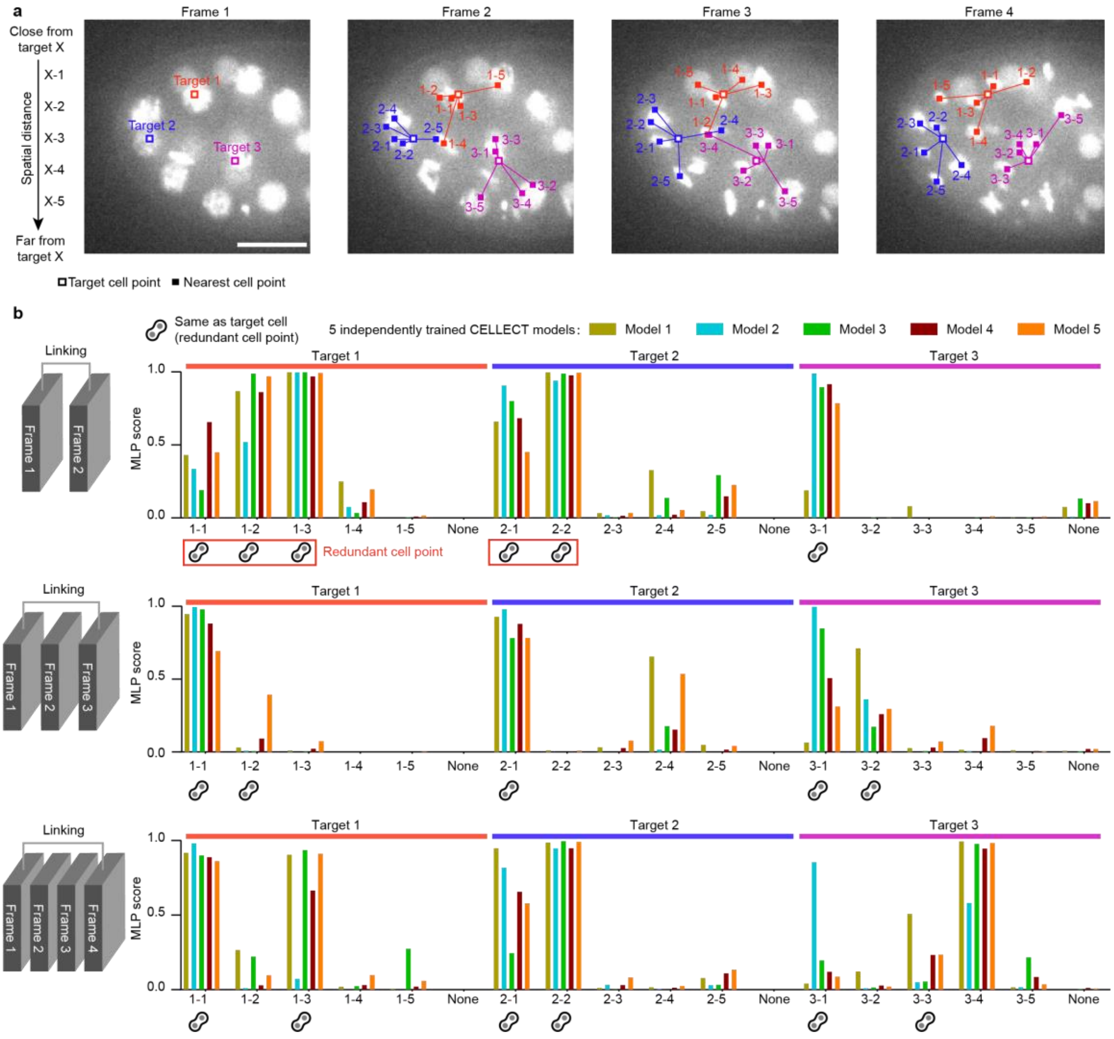

Supplementary Figure 14 | Evaluation of the reproducibility of cell linking during different imaging frame rate. To evaluate the reproducibility of CELLECT's cell linking under different imaging frame rate, we selected 3 representative target cells (Target #1–3) from mskcc-confocal dataset #2 and selected 5 nearest spatial neighbors at frames t+1, t+2, and t+3 for testing. **a**, Location of the target cells at time t (frame 1) and the locations of candidate cells across the 3 subsequent frames. **b**, Similarity scores predicted by 5 independently trained CELLECT models for the 5 candidates. To ensure consistent input across models, redundant point removal was not applied intentionally, resulting in occasional duplicate redundant cell points (e.g., Target #1 and #2 at frame 1 linked to frame 2). This configuration increases linking difficulty by expanding the temporal gap, thereby simulating more complex multi-frame association scenarios. While correct links often correspond to the nearest candidate in adjacent-frame conditions, longer intervals such as frame 1 to frame 3 or frame 4 require the model to rely more on embedding features for accurate decisions. The results demonstrate that CELLECT produces consistent linking performance across different frame intervals. However, under low imaging frame rate and in the presence of cell divisions (e.g., frame 1 linked to frame 4), a slight drop in accuracy is observed. All 5 models were independently trained on mskcc-confocal dataset #3. Scale bar: 10 µm.

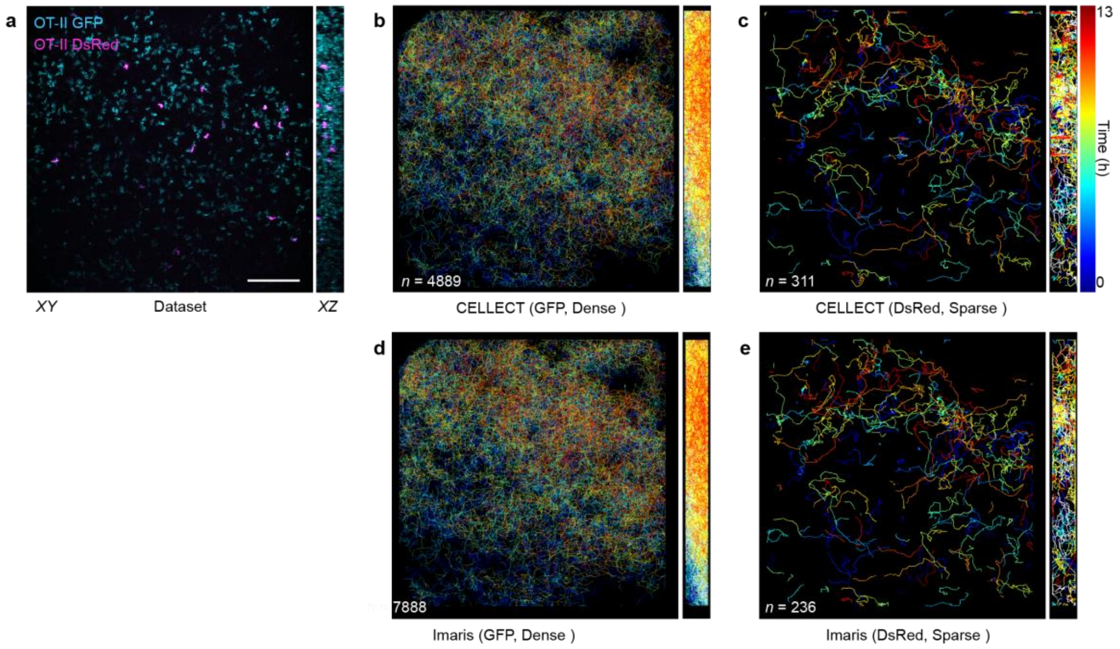

Supplementary Figure 15 | Cell tracking for dual-color labeled T cells. a, Dual-color labeled T cell imaging dataset. T cells were labeled with both DsRed and GFP at a 1:5 ratio. Sparsely labeled cells generally appeared in the same locations as densely labeled cells. **b-c**, Tracking result of both dense and sparse imaging channel datasets generated by CELLECT. **d-e**, Tracking result of both dense and sparse imaging channel dataset generated by Imaris. All imaging data were acquired using 2pSAM microscopy of mouse lymph nodes, consistent with the dataset used in **Fig. 3**. CELLECT results were generated using the model trained on sequence #3 of the mskcc-confocal dataset. Scale bars: 100 µm.

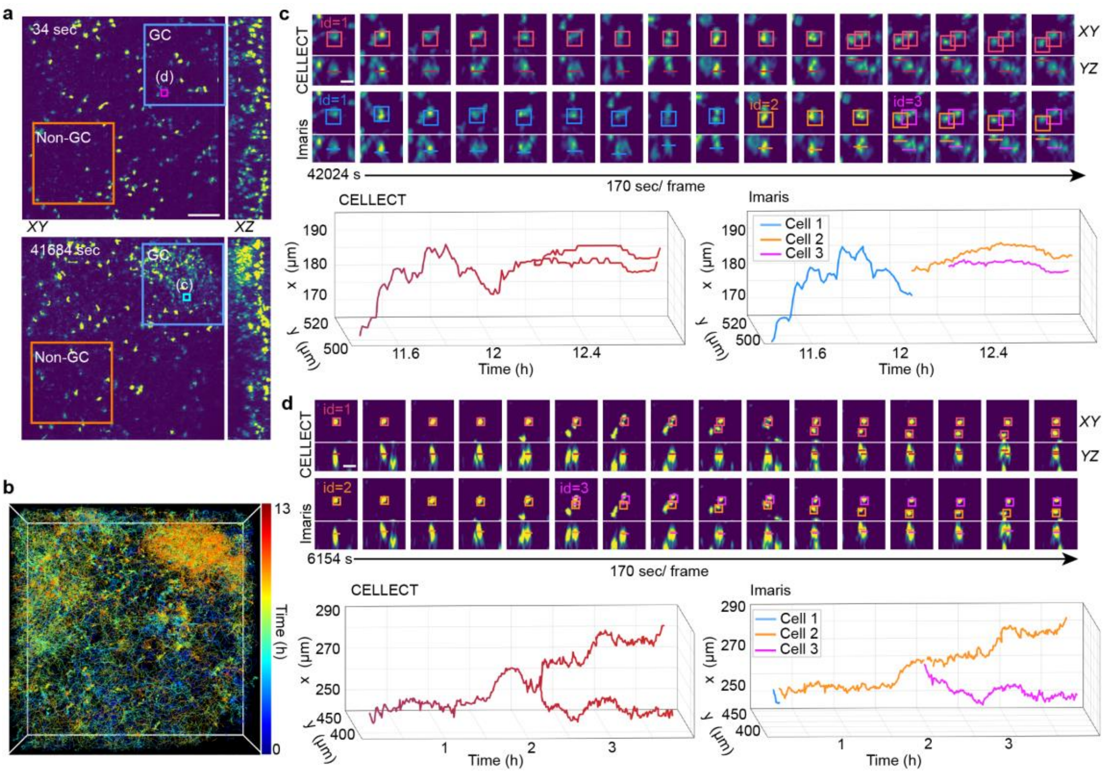

Supplementary Figure 16 | Comparisons of the tracking results of B-cell dynamics obtained by CELLECT and Imaris. a, Germinal center (GC) dataset. **b**, All tracking trajectories. **c-d**, Display of the cell division process for 2 cell samples and the tracking trajectories obtained by both methods. In some cases, Imaris shows multiple cells due to its insufficient detection of cell divisions, so to make a comparison, we reconstructed the full division process by stitching together the trajectories of nearby cells. All data were acquired using 2pSAM imaging of B cells in mouse lymph nodes, consistent with the dataset used in **Fig. 3**. CELLECT results were generated using the model trained on sequence #3 of the mskcc-confocal dataset. Scale bars: 100 µm (**a**), 10 µm (**c**), 20 µm (**d**).

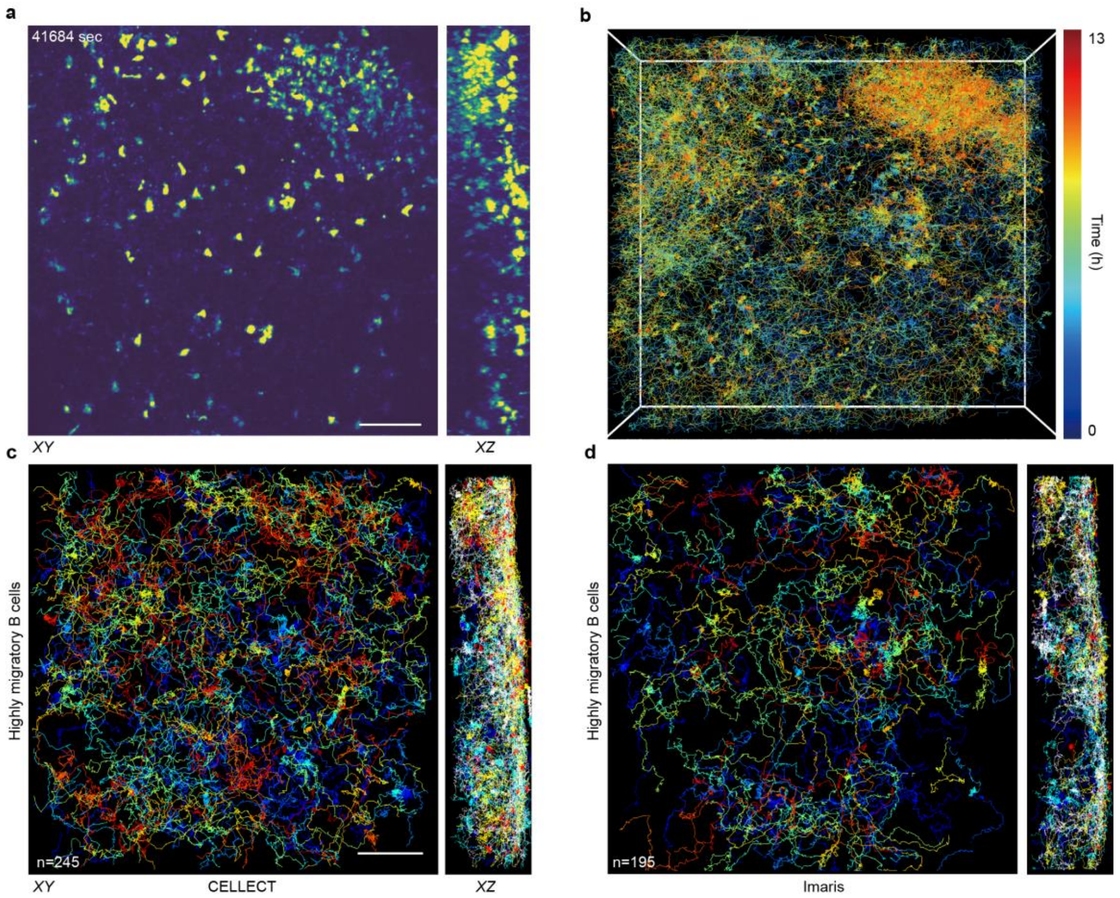

Supplementary Figure 17 | Extracted traces of highly migratory B cells. a, Dataset capturing B cell dynamics during germinal center (GC) formation. **b**, 3D cell tracking results generated by CELLECT. **c-d**, Trajectories of extraction of highly migratory B cells (defined as cells moving more than 50 μm in over an hour) via CELLECT (**c**) and Imaris (**d**), respectively. All imaging data were acquired using 2pSAM of B cells in mouse lymph nodes during GC formation, consistent with the dataset used in **Fig. 3**. CELLECT results were generated using the model trained on sequence #3 of the mskcc-confocal dataset. Scale bars: 100 μm.

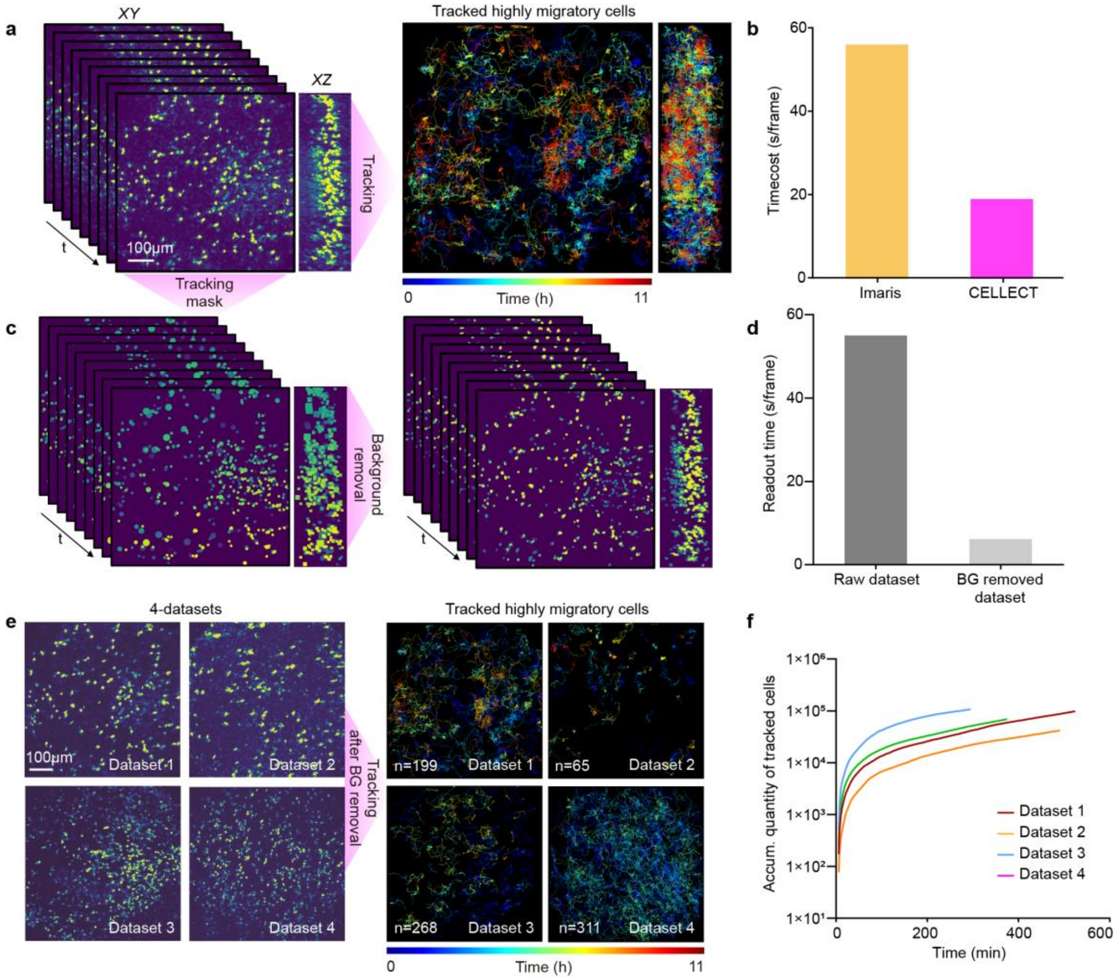

Supplementary Figure 18 | Tracking performance of CELLECT on terabyte-level imaging datasets of B-cell dynamics. a, Example MIPs from the data sequence and corresponding cell tracking results. **b**, Comparison of the running speed between CELLECT and Imaris. **c**, Background removal based on our tracking results, which reduces data storage size and subsequent reading time. **d**, Comparison of reading times after background removal. **e**, Display of long-distance migration trajectories across all 4 datasets. **f**, Integral representation of cell counts in the 4 datasets. All data were acquired using 2pSAM imaging of B-cell dynamics in mouse lymph nodes. These datasets are distinct from the one shown in **Fig. 3**, but were collected under identical imaging conditions and protocols. The total data volume exceeds 4 TB. CELLECT results were generated using the model trained on sequence #3 of the mskcc-confocal dataset. Scale bars: 100 μm.

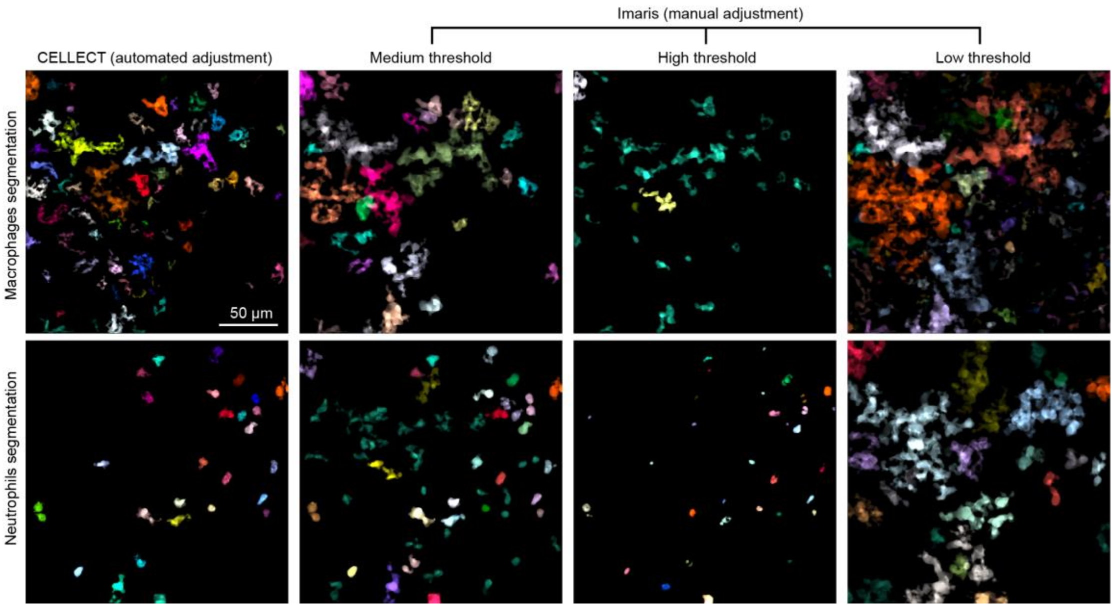

Supplementary Figure 19 | Influence of the intensity threshold on the segmentation results of Imaris with comparisons to CELLECT. The segmentation via CELLECT does not require threshold adjustment (left), ensuring more consistent and unbiased results interpretation. In contrast, the segmentation performance of the signal intensity-based algorithm in Imaris is highly sensitive to thresholding parameters, which can lead to variable outcomes from the same dataset. The data used here were acquired via 2pSAM imaging of neutrophils, macrophages, and bacteria in mouse spleen, consistent with the dataset shown in **Fig. 4**. CELLECT results were generated using the model trained on sequence #3 of the mskcc-confocal dataset. Scale bars: 50 μm.

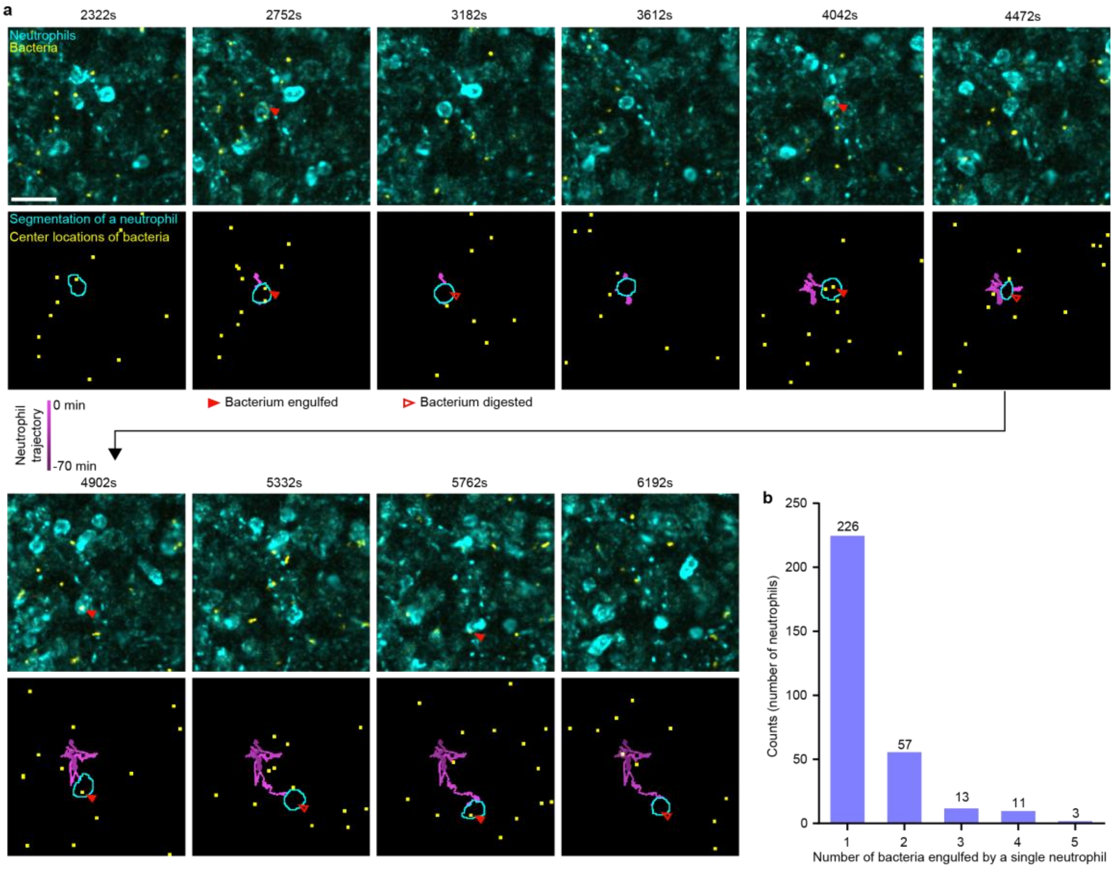

Supplementary Figure 20 | Continuous tracking and segmentation of single neutrophil chasing and engulfing bacteria. a, The upper row shows a 2-channel sequential image dataset of bacteria and neutrophils over a 70-minute time window, while the lower row shows representative tracking and segmentation of a single neutrophil pursuing bacteria. The segmented neutrophil is outlined with a solid cyan line, and trajectory is shown as a purple gradient line. Bacterial center positions are marked yellow. Engulfment and digestion events are indicated by red-filled and red-outline triangles, respectively. **b**, Histogram of the number of bacterial engulfment events per neutrophil. The data used here were acquired via 2pSAM imaging of neutrophils, macrophages, and bacteria in mouse spleen, consistent with the dataset shown in **Fig. 4**. CELLECT results were generated using the model trained on sequence #3 of the mskcc-confocal dataset. Scale bars: 20 μm

Supplementary Table 1 | Quantitative results on mskcc-confocal and nih-ls dataset [2,3]. For descriptions of the error metrics, see **Supplementary Fig. 6**. All values represent absolute errors normalized per 1,000 ground-truth (GT) edges; best value bold.

	FP	FN	IS	FP-D	FN-D	Division	Sum
Mskcc-confocal 270 frames							
StarryNite*	7.9	13	0.62	0.58	1.2	1.8	24
Linajea*	3.6	5.5	0.062	0.89	0.26	1.2	10.3
Linajea+csc+sSVM*	3.7	5.6	0.046	**0.053**	**0.4**	**0.46**	9.6
CELLECT	**1.26**	**3.56**	**0.013**	0.076	0.41	0.49	**5.32**
Nih-ls 270 frames							
StarryNite*	22	18	2.4	0.66	1.6	2.2	45
Linajea*	12	6.5	0.46	1.5	**0.4**	1.86	21
Linajea+csc+sSVM*	13	5.3	0.59	**0.20**	0.49	**0.69**	20
CELLECT	**6.37**	**3.68**	**0.070**	0.777	1.14	1.92	**12.04**

* The values are obtained from the previous report (Hirsch, P. et al. *Medical Image Computing and Computer Assisted Intervention 2022,* 25–35 (2022).

Supplementary Table 2 | Quantitative results for each fold in the cross-validation experiments on mskcc-confocal and nih-ls data. For descriptions of the error metrics, see **Supplementary Fig. 6**. All values represent absolute errors normalized per 1,000 ground-truth (GT) edges.

Train-id	Test-id	FP	FN	IS	FP-D	FN-D	Division	Sum
Mskcc-confocal 270 frames								
1	2	1.38	2.13	0.039	0.099	0.48	0.58	4.13
	3	0.9	3.62	0.039	0.039	0.26	0.30	4.86
2	1	1.32	5.33	0.0	0.097	0.50	0.60	7.25
	3	1.89	5.20	0.0	0.14	0.33	0.47	7.56
3	1	1.12	2.75	0.0	0.058	0.39	0.40	4.32
	2	0.95	2.34	0.0	0.02	0.50	0.52	3.81
Mean		1.26	3.56	0.013	0.076	0.41	0.49	5.32
Nih-ls 270 frames								
1	2	4.71	4.36	0.018	0.712	1.57	2.28	11.37
	3	6.14	6.38	0.049	0.5	0.77	1.27	13.84
2	1	2.44	5.21	0.064	0.614	1.67	2.28	9.99
	3	15.41	1.37	0.129	1.565	0.364	1.93	18.84
3	1	3.43	3.71	0.085	0.678	1.01	1.69	8.91
	2	6.09	1.05	0.074	0.591	1.46	2.05	9.27
Mean		6.37	3.68	0.070	0.777	1.14	1.92	12.04

Supplementary Table 3 | Input and output tensor dimensions for each module.

Module	Input Size	Description	Output Size	Description
3D U-Net	2 × 256 × 256 × 32	Two consecutive 3D image frames	4 × 256 × 256 × 32	Confidence map (background/embryo /polar body/size)
			2 × 256 × 256 × 32	Division probability map (2-classes)
			64 × 256 × 256 × 32	Embedding map
CEN	6 × 256 × 256 × 32	Two frames + confidence map	5 × 256 × 256 × 32	Confidence map (center-enhanced, 4 levels + background)
Intra-frame MLP	64 × 1	Current cell embedding	6 × 1	5 candidates + ‘None’ scores
	64 × 5	Embeddings of 3candidates		
	3 × 5	Spatial distance to candidates		
	6 × 5	Difference and ratio of size and spatial distance		
	6 × 5	Difference and ratio of division		
Inter-frame MLP	64 × 1	Current cell embedding	6 × 1	5 candidates + ‘None’ scores
	64 × 5	Embeddings of 5 candidates		
	3 × 5	Spatial distance to candidates		
	6 × 5	Difference and ratio of size and spatial distance	2 × 1	Division probability
	6 × 5	Difference and ratio of division probability		

Supplementary Table 4 | Quantitative ablation study of individual modules in the CELLECT framework. We assessed the contributions of the Center Enhancement Network (CEN), intra-frame MLP, and inter-frame MLP by progressively enabling each. Tracking Accuracy is defined as the proportion of uninterrupted, error-free trajectories over 50 to 200 frames[2] and Error Links refer to the total tracking errors (false negatives, false negative divisions, and identity switches). In ablated models, nearest-neighbor matching replaced the inter-frame MLP, and redundancy filtering was disabled without the intra-frame MLP. Removing CEN resulted in noisy center proposals due to unenhanced confidence maps. Model was trained on mskcc-confocal #3 and evaluated #2.

3D U-Net	CEN	Intra-frame MLP	Inter-frame MLP	Tracking Accuracy[2] Length (frames)				Error Links (FN+FND+IS)
				50	100	150	200	
Yes	No	No	No	0.76	0.42	0.13	0.02	382
Yes	Yes	No	No	0.84	0.55	0.21	0.10	219
Yes	Yes	Yes	No	0.85	0.57	0.23	0.10	210
Yes	Yes	Yes	Yes	0.94	0.84	0.61	0.32	138

Supplementary Video 1 | Confidence map generated by CELLECT on a dataset of membrane-labeled cells. We captured the dynamic migration of neutrophils in the spleen using intravital imaging with scanning light-field microscope[4] (left). From this dataset, we generated and visualized confidence maps with and without CEN using CELLECT (middle and right). The confidence maps generated by CELLECT provide a clearer visualization of cell regions and their movement dynamics. The standard confidence map (without CEN) captures a broader area of the cell regions (middle), while the center-enhanced confidence map (with CEN) highlights only a small, concentrated portion of the cell centers (right). CELLECT results were generated using the model trained on sequence #3 of the mskcc-confocal dataset.

Supplementary Video 2 | Efficient cell tracking of CELLECT on *C. elegans* dataset compared with Linajea. Left: MIP of the publicly available dataset (Mskcc-confocal) showing the *C. elegans* embryo development process. Middle: Visualization of cell tracking results using CELLECT. Right: Visualization of cell tracking results using Linajea. The initial 4 seed cells are marked with distinct 4 colors. Differentiated cells are assigned the same color as their mother cell, illustrating lineage tracing capability. CELLECT results were generated using the model trained on sequence #3 of the mskcc-confocal dataset.

Supplementary Video 3 | 3D cell tracking of large-scale B cells during the formation of germinal center. Upper left: Orthogonal MIPs of B-cells dynamics in mouse lymph node during germinal center formation captured by 2pSAM. Upper middle: Short-term traces of B-cell migration over the last 10 minutes. Upper right: Continuous traces of B-cell migration. Bottom: Visualization of continuous tracking of a single cell dividing into 2 cells, as obtained by CELLECT and Imaris, respectively. CELLECT results were generated using the model trained on sequence #3 of the mskcc-confocal dataset.

Supplementary Video 4 | Continuous tracking of neural activities at single-cell resolution in *Drosophila* brain during tissue deformation. **a**, MIP of neural activities in *Drosophila* brain with tracking results obtained by CELLECT, Imaris, and TrackMate-Weka for comparisons. **b**, Evaluation of long-term tracking performance by tracking cells from the first frame. Fully tracked cells are shown in green, while partially tracked cells are shown in red. **c**, Heatmap of the neural activities of tracked cells over time. **d**, Representative results of a single neuron tracked by 3-different methods (CELLECT, Imaris and TrackMate-Weka) for comparisons. **e**, Demonstration of multiple cells tracked by CELLECT. 5 cells are depicted with 5 different colors. Newly detected cells after the first frame are not visualized or analyzed in this study. The calcium traces shown in (**c-e**) are normalized. The areas corresponding to (**d**) and (**e**) are depicted with blue and purple squares in the MIP shown in (**a**). CELLECT results were generated using the model trained on sequence #3 of the mskcc-confocal dataset.

References

1. Santella, A., Du, Z. & Bao, Z. A semi-local neighborhood-based framework for probabilistic cell lineage tracing. *BMC Bioinform.* **15**, 217–217 (2014).

2. Hirsch, P. *et al.* Tracking by weakly-supervised learning and graph optimization for whole-embryo C. elegans lineages. *Medical Image Computing and Computer Assisted Intervention (MICCAI)* 25–35 (2022).

3. Moyle, M. W. *et al.* Structural and developmental principles of neuropil assembly in C. elegans. *Nature* **591**, 99–104 (2021).

4. Wu, J. *et al.* Iterative tomography with digital adaptive optics permits hour-long intravital observation of 3D subcellular dynamics at millisecond scale. *Cell* **184**, 3318-3332. e17 (2021).
